# Dataset on the effects of self-confidence, motivation and anxiety on Indonesian students’ willingness to communicate in face-to-face and digital settings

**DOI:** 10.1016/j.dib.2020.105774

**Published:** 2020-05-27

**Authors:** Herri Mulyono, Regitha Saskia

**Affiliations:** University of Muhammadiyah, Prof. DR HAMKA, Jakarta, Indonesia

**Keywords:** Willingness to communicate, self-confidence, anxiety, motivation, face-to-face (F2F) and digital environments

## Abstract

This dataset presents the relationship of three affective variables (i.e. self-confidence, motivation and anxiety) with English as a foreign language (EFL) students’ willingness to communicate (WTC) in face-to-face (F2F) and digital settings. Students’ WTC is measured in F2F settings both inside and outside the classroom. A non-probability convenience sampling technique was employed to target the study participants. A total of 458 Indonesian EFL students completed a WTC questionnaire designed by Lee and Hsieh [1], of which 436 responses were analysed. Statistical analyses (i.e. correlation and regression calculations) were carried out on the quantitative data. The dataset is significant for the Ministry of Education and Culture (MoEC), school principals, and EFL teachers, in designing curriculum and instructional activities that enhance EFL students’ willingness to communicate in the target language inside and outside classrooms, and in the digital environment. The dataset is also useful for further research evaluating students’ WTC in digital settings, and addressing the issue of students’ WTC in written and oral communications.

Specifications table

**Table:** 

**Subject**	Education
**Specific subject area**	Applied linguistics, language education
**Type of data**	Table
**How data were acquired**	A survey method was employed to collect the data
**Data format**	Raw Analysed Filtered
**Parameters for data collection**	Data on students’ levels of self-confidence, motivation and anxiety, and their WTC, were collected using a questionnaire designed by Lee and Hsieh [1].
**Description of data collection**	A total of 458 Indonesian EFL students completed Lee and Hsieh's [1] original questionnaire. The questionnaire is provided as a supplementary file. After a screening process, 436 responses were analysed; more participants were female (*N*=323, 74.08%) than male (*N*=113, 25.92%); the majority were university students (*N*=259, 59.40%), followed by upper-secondary school students (*N*=96, 22.02%) and lower-secondary school students (*N*=81, 18.58%).
**Data source location**	Indonesia's main islands, including Sumatera, Java, Sulawesi, Kalimantan, and one small island, East Nusa Tenggara.
**Data accessibility**	The data are available in Mendeley Data: https://data.mendeley.com/datasets/hccbrpzw7k/3

## Value of the data

The dataset describes EFL students’ WTC in F2F and digital settings across secondary schools and universities in Indonesia. The data are useful in providing valuable information to policy makers, administrative staff and EFL teachers at schools and universities, regarding the role of self-confidence, motivation and anxiety in promoting students’ WTC in F2F and digital settings.

The data benefits the Ministry of Education and Culture (MoEC), school principals and EFL teachers, in designing curriculum and instructional activities that enhance EFL students’ willingness to communicate in the target language inside and outside classrooms, and in the digital environment. Particularly, the data will help EFL teachers to address learning problems related to students’ self-confidence, motivation and anxiety, which affect their WTC.

The data are useful for educational researchers who investigate the role of affective variables influencing EFL students’ WCT in three different settings (i.e. inside the classroom, outside the classroom and in the digital environment), and those who examine EFL students’ WTC in digital written and oral communication contexts. The data also can be re-used to validate Lee and Hsieh's WTC instrument [1].

## Data Description

The dataset was gathered using a survey method. A five-point Likert scale questionnaire proposed by Lee and Hsieh [1] was adopted. The questionnaire consisted of 33 items classified into four affective variables and second-language (L2) WTC environments. The affective variables comprised self-confidence (six items), speaking anxiety (six items), motivation (four items) and grit (five items). The “self-confidence”, “speaking anxiety” and “motivation” sub-scales adopted a five-point scale, ranging from “Strongly disagree” (1) to “Strongly agree” (5), while the “grit” subscale ranged from “Not like me at all” (1) to “Very much like me” (5). All items in L2 WTC environments used a five-point scale, ranging from “Strongly disagree” (1) to “Strongly agree” (5). The questionnaire is provided as a supplementary file.

## Experimental Design, Materials and Methods

A non-probability convenience sampling technique was employed to target the study participants. An online version of Lee and Hsieh's [1] questionnaire was developed to facilitate administration, to target a wide range of participants, and to allow automated data collection and tabulation [2,3]. A survey link was posted in several EFL student online groups and pages of social media platforms such as Facebook, Twitter and WhatsApp. A total of 458 Indonesian EFL students completed Lee and Hsieh's original questionnaire [1]. Prior to the data analyses, the data collected were scrutinised for missing values and outliers. Using a box plot, 22 responses were identified as outliers and thus were deleted [4]. The remaining 436 data showed that more participants were female (*N*=323, 74.08%) than male (*N*=113, 25.92%); the majority were university students (*N*=259, 59.40%), followed by upper-secondary school students (*N*=96, 22.02%) and lower-secondary school students (*N*=81, 18.58%). The remaining 436 questionnaires’ data were then analysed.

Reliability analysis of the questionnaire was conducted by evaluating the Cronbach's α. As shown in Table 1, the sub-scales of “self-confidence”, “speaking anxiety”, “F2F WTC inside classroom”, “F2F outside classroom”, and “WTC in a digital environment” were reported to possess high levels of internal consistency, while the “Motivation” sub-scale was marginally reliable, and the “grit” sub-scale had unacceptably low reliability [4]. Gliner, Morgan and Leech [5] argue that a measure with a marginal level of reliability coefficient (e.g. above 0.60) is still acceptable for use as a data collection instrument. Consequently, the “Motivation” sub-scale was used but the “Grit” sub-scale was excluded from further analyses.

**Table 1:** Reliability and descriptive data

Constructs	Items	*Mean*	*SD*	*Cronbach's α*
Self-confidence	1	3.95	0.72	0.84
	2	4.11	0.68	
	3	3.76	0.73	
	4	3.49	0.81	
	5	3.89	0.73	
	6	3.68	0.75	
Speaking anxiety	1	3.59	1.04	0.88
	2	3.32	1.08	
	3	3.46	1.06	
	4	2.98	1.09	
	5	3.21	1.13	
	6	3.11	1.23	
Motivation	1	4.07	0.72	0.69
	2*	4.07	1.04	
	3	4.22	0.72	
	4	4.43	0.66	
Grit	1	3.85	0.90	0.36
	2*	2.45	0.93	
	3	3.88	0.84	
	4*	2.64	1.03	
	5*	2.74	1.06	
F2F WTC inside the classroom	1	4.15	0.82	0.87
	2	4.07	0.81	
	3	4.01	0.88	
	4	3.85	0.91	
F2F WTC outside the classroom	1	4.04	0.89	0.88
	2	3.83	0.97	
	3	4.05	0.85	
	4	3.77	0.96	
WTC in a digital environment	1	4.28	0.845	0.82
	2	4.25	0.847	
	3	4.18	0.866	
	4	3.98	1.01	

*Note.* * items were reverse coded; SD = standard deviation

A correlation analysis was performed on the data and the results are shown in Table 2 below:

**Table 2:** Correlations among variables

Variable	1	2	3	4	5	6	7	8	9
Gender	1	0.137**	0.221**	-0.05	0.022	0.092	0.047	0.082	0.048
Age		1	0.865**	0.115*	0.008	0.085	0.206**	0.132**	0.064
Level of education			1	0.083	0.092	0.072	0.192**	0.134**	0.104*
Self-confidence				1	-0.340**	0.412**	0.442**	0.369**	0.335**
Speaking anxiety					1	-0.373**	-0.152**	-0.060	-0.040
Motivation						1	0.491**	0.407**	0.403**
F2F WTC inside the classroom							1	0.717**	0.503**
F2F WTC outside the classroom								1	0.584**
WTC in a digital environment									1

*Note.* *p < 0.05;^⁎⁎^*p* < 0.01

The results of the correlation calculation shown in Table 2 above suggest that F2F WTC inside the classroom was statistically associated with age (*r=0.206, p<0.01*), educational level (*r=0.192, p<0.01*), self-confidence (*r=0.442, p<0.01*) and motivation (*r=0.491, p<0.01*). The result also shows that F2F WTC inside the classroom had a negative correlation with speaking anxiety (r= -0.152, p<0.01). Furthermore, age (*r=0.132, p<0.01*) and level of education (*r=0.134, p<0.01*), and two affective variables, i.e. self-confidence (*r=0.369, p<0.01*) and motivation (*r=0.407, p<0.01*), were correlated with F2F WTC outside the classroom. Within a digital environment, students’ WTC significantly correlated with education level (r=0.104, p<0.05), self-confidence (*r=0.335, p<0.01*) and motivation (*r=0.403, p<0.01*).

Statistical assumptions in the application of multiple regression were evaluated, to ensure the robustness of the regression model, in aspects including sample size, outlier issues, multicollinearity, and normal distribution of the residuals [4,6]. The dataset met the assumptions, except for the issue of normality in regression model 1, where the regression analysis was performed on the demographic aspects without including the affective variables [1]. Regression model 1 for all WTC settings (i.e. inside, outside classrooms and in the digital environment) suggested non-normal distribution of the data (*p<0.01*). Hence, regression model 2 was developed by involving all demographic aspects and affective variables; the result indicated that the data for F2F WTC and WTC in a digital environment were normally distributed (*p>0.01*). The model also showed that gender, age and level education did not significantly correlate with F2F WTC inside the classroom (*p>0.01*). However, three affective variables significantly correlated with students’ WTC inside classroom: self-confidence (*B=0.269, p<0.01*); speaking anxiety (*B=0.089, p<0.05*); and motivation (*B=0.39, p<0.01*). The variables also significantly influenced F2F WTC outside the classroom (self-confidence, *B=0.277, p<0.01*; speaking anxiety, *B=0.160, p<0.01*; and motivation, *B=0.342, p<0.01*), and WTC in a digital environment (self-confidence, *B=0.244, p<0.01*; speaking anxiety, *B=0.168, p<0.01*; and motivation, *B=0.364, p<0.01*). Among the three affective variables, motivation was the greatest influence on students’ F2F WTC, and their WTC in a digital environment.

## Declaration of Competing Interest

The authors declare that they have no known competing financial interests or personal relationships which have, or could be perceived to have, influenced the work reported in this article.

## Appendix Supplementary materials

Image, application 1

Image, application 2
