# Supplementary material for: Dataset on the effects of self-confidence, motivation and anxiety on Indonesian students’ willingness to communicate in face-to-face and digital settings
Source: Data Brief. 2020 May 27;31:105774. doi: 10.1016/j.dib.2020.105774 (PMC7286956; doi:10.1016/j.dib.2020.105774)
Supplement: Supplementary file 1 [file mmc1.docx]

**Supplementary article**

Lee and Hsieh’s (2019) WTC questionnaire

**Part 1. Affective variables**

| Self-confidence | Strongly agree | Agree | Neutral | Disagree | Strongly disagree |
| --- | --- | --- | --- | --- | --- |
| 1. I am confident I can use English vocabulary and expressions that I learned to interact with my teacher or friends. |  |  |  |  |  |
| 1. I am confident I can talk about myself in English using sentences. |  |  |  |  |  |
| 1. I am confident I can talk about what I did last weekend using English sentences. |  |  |  |  |  |
| 1. I am confident I can do well in oral presentation in English. |  |  |  |  |  |
| 1. I am confident I can order a meal in English at a restaurant. |  |  |  |  |  |
| 1. I am confident I can ask for and give directions for location in English. |  |  |  |  |  |

| L2 anxiety | Strongly agree | Agree | Neutral | Disagree | Strongly disagree |
| --- | --- | --- | --- | --- | --- |
| 1. I feel nervous when I speak in English in front of other students. |  |  |  |  |  |
| 1. I feel anxious if I am asked a question by my teacher. |  |  |  |  |  |
| 1. When speaking in English, I can get so nervous that I forget things that I know. |  |  |  |  |  |
| 1. I feel nervous when I am called upon to perform a task in English. |  |  |  |  |  |
| 1. Even if I am well prepared for English class, I feel anxious about it. |  |  |  |  |  |
| 1. I am afraid that the other students will laugh at me when I speak English. |  |  |  |  |  |

| Motivation | Strongly agree | Agree | Neutral | Disagree | Strongly disagree |
| --- | --- | --- | --- | --- | --- |
| 1. I actively think about what I have learned in my English class. |  |  |  |  |  |
| 1. I don't really have a great desire to learn English. |  |  |  |  |  |
| 1. I find learning English very enjoyable. |  |  |  |  |  |
| 1. English is an important subject to me in my college program. |  |  |  |  |  |

| Grit | Very much like me | Mostly like me | Somewhat like me | Nor much like me | Not like me at all |
| --- | --- | --- | --- | --- | --- |
| 1. Setbacks don't discourage me. |  |  |  |  |  |
| 1. I have been obsessed with a certain idea or project for a short time but later lost interest. |  |  |  |  |  |
| 1. I am a hard worker. |  |  |  |  |  |
| 1. I often set a goal but later choose to pursue a different one. |  |  |  |  |  |
| 1. I have difficulty maintaining my focus on projects that take more than a few months to complete. |  |  |  |  |  |

**Part 2. WTC in contexts**

| WTC inside classroom | Definitely willing | Probably willing | Perhaps willing | Probably not willing | Definitely not willing |
| --- | --- | --- | --- | --- | --- |
| 1. When you are given a chance to talk freely in an English class. |  |  |  |  |  |
| 1. When you have a chance to talk in front of the other students in an English class. |  |  |  |  |  |
| 1. When you have a group discussion in an English class. |  |  |  |  |  |
| 1. When you have a chance to make a presentation in front of a large group. |  |  |  |  |  |

| WTC outside classroom | Definitely willing | Probably willing | Perhaps willing | Probably not willing | Definitely not willing |
| --- | --- | --- | --- | --- | --- |
| 1. When you find your friend standing in front of you in a line. |  |  |  |  |  |
| 1. When you find your acquaintance standing in front of you in a line. |  |  |  |  |  |
| 1. When you have a discussion with a small group of friends. |  |  |  |  |  |
| 1. When you have a chance to talk as part of a small group of strangers. |  |  |  |  |  |

| WTC in digital context | Definitely willing | Probably willing | Perhaps willing | Probably not willing | Definitely not willing |
| --- | --- | --- | --- | --- | --- |
| 1. When you chat with non-native speakers of English (e.g., Korean, Japanese, Chinese) on Facebook. |  |  |  |  |  |
| 1. When you chat with native speakers of English (e.g., American, Australian, British) on Facebook. |  |  |  |  |  |
| 1. When you post comments to a foreign friend's wall on Facebook. |  |  |  |  |  |
| 1. When you talk to other users of English while playing games |  |  |  |  |  |

References:

Lee, J. S., & Hsieh, J. C. (2019). Affective variables and willingness to communicate of EFL learners in in-class, out-of-class, and digital contexts. *System*, *82*, 63–73.
